# 高效液相色谱-电喷雾电离串联质谱法测定克唑替尼原料药中的基因毒性杂质

**DOI:** 10.3724/SP.J.1123.2025.04017

**Published:** 2025-11-08

**Authors:** Xiaobing ZHAN, Yi QIN, Ying YAN, Jie ZHOU, Longshan ZHAO

**Affiliations:** 1.沈阳药科大学，辽宁 沈阳 110016; 1. Shenyang Pharmaceutical University，Shenyang 110016，China; 2.江苏万邦生化医药集团有限责任公司，江苏 徐州 221000; 2. Jiangsu Wanbang Biopharmaceuticals，Xuzhou 221000，China

**Keywords:** 非小细胞肺癌, 克唑替尼原料药, 基因毒性杂质, 高效液相色谱-电喷雾电离串联质谱, **n**on-small cell lung cancer, the bulk drug of crizotinib, genotoxic impurity, high performance liquid chromatography-electrospray ionization tandem mass spectrometry （HPLC-ESI-MS/MS）

## Abstract

建立了一种高效液相色谱-电喷雾电离串联质谱（HPLC-ESI-MS/MS）方法，用于测定克唑替尼原料药中潜在基因毒性杂质（WHT1408-Q2H）的含量。实验中利用Agilent Eclipse XDB C8色谱柱（150 mm×4.6 mm，3.5 µm），以0.1%甲酸水-0.1%甲酸乙腈为流动相，流速为0.4 mL/min，梯度洗脱，柱温为40 ℃，进样量5 μL；质谱部分采用电喷雾正离子（ESI^+^）多反应监测（MRM）扫描模式，杂质WHT1408-Q2H的［M+H］^+^母离子为*m*/*z* 205.3，碎片离子为*m*/*z* 121.0。结果显示，所建立的方法专属性良好，WHT1408-Q2H在2~40 ng/mL范围内呈现出良好的线性关系，相关系数（*r*）为0.999 9；检出限为0.396 9 ng/mL，定量限为1.984 6 ng/mL；在低、中、高3个水平下的回收率为95.6%~102.7%，RSD为0.4%~0.7%。应用该方法对3批克唑替尼原料药样品中的WHT1408-Q2H进行测定，3批样品中均未检出WHT1408-Q2H，表明当前生产工艺可有效控制该基因毒性杂质的含量。该方法专属性强，灵敏度高，操作简单，适用于克唑替尼原料药中基因毒性杂质WHT1408-Q2H的严格质量控制。依据M7基因毒性杂质指导原则，该方法能够准确定量测定痕量的遗传毒性杂质，将进一步确保药物符合监管要求并保障药物安全。未来该分析方法将扩展到其他治疗药物中类似的遗传毒性杂质评估，从而推进药物杂质分析和控制。

## Abstract (en)

A method based on high performance liquid chromatography-electrospray ionization tandem mass spectrometry （HPLC-ESI-MS/MS） was developed for the determination of the potential genotoxic impurity （WHT1408-Q2H） in the bulk drug of crizotinib. An Agilent Eclipse XDB C8 chromatographic column （150 mm×4.6 mm， 3.5 µm） was used for chromatographic separation. The mobile phases were 0.1% formic acid aqueous solution and 0.1% formic acid acetonitrile solution at a flow rate of 0.4 mL/min. The column temperature was maintained at 40 ℃ and the sample size was 5 μL. In the mass spectrometry section， the electrospray positive ion （ESI^+^） mode with multiple reaction monitoring （MRM） scanning was adopted. The accurate mass of the ［M+H］^+^ parent ion of WHT1408-Q2H was *m*/*z* 205.3， and the accurate mass of the extracted fragment ion was *m*/*z* 121.0. The results of methodological validation demonstrated that the established method exhibited excellent specificity. The peak area and mass concentration of WHT1408-Q2H exhibited a good linear relationship within the range of 2-40 ng/mL， with a correlation coefficient （*r*） of 0.999 9. The limit of detection （LOD） and limit of quantitation （LOQ） for WHT1408-Q2H were 0.396 9 ng/mL and 1.984 6 ng/mL， respectively. The recoveries of WHT1408-Q2H at low， medium， and high levels were in the range of 95.6%-102.7%， while the relative standard deviations （RSDs） were between 0.4% and 0.7%. Finally， the proposed method was successfully applied to analyze three independent batches of the bulk drug of crizotinib. The results revealed that WHT1408-Q2H was not detected in all samples， indicating that the current production process can effectively control the content of this genotoxic impurity. In conclusion， the developed HPLC-ESI-MS/MS method is highly specific， sensitive， and simple， making it suitable for the stringent quality control of WHT1408-Q2H in the bulk drug of crizotinib. According to the M7 guideline on genotoxic impurities， this method is capable of accurately quantifying trace amounts of genotoxic impurities and will further ensure compliance with regulatory requirements and safeguard drug safety. Future applications may extend this analytical framework to similar genotoxic impurities assessment in other therapeutic compounds， thereby advancing the field of pharmaceutical impurity profiling and control.

肺癌是世界范围内最常见的癌症之一，根据癌细胞的形态及外观大小可分为非小细胞癌，小细胞癌和混合小细胞癌。其中非小细胞肺癌（NSCLC）是最常见的形式，占所有肺癌的85%^［1，2］^。研究表明，治疗NSCLC的靶点有表皮因子生长受体（EFGR）、间质表皮转化因子（MET）、渐变性淋巴瘤激酶（ALK）等^［3-5］^。克唑替尼是第一代被研究的ALK和MET抑制剂，对于既往接受过治疗的ALK重排晚期非小细胞肺癌患者，克唑替尼优于标准化疗，能够延长患者无进展生存期，改善生活质量^［6，7］^。

基因毒性杂质（genotoxic impurity， GTI）是一类能够直接或间接损伤DNA、诱发基因突变或染色体畸变的化合物，即使在痕量水平也可能对患者构成潜在的致癌风险^［8］^。因此，各国药品监管机构（如人用药品技术要求国际协调理事会（ICH）、美国食品药物管理局（FDA）、欧洲药品管理局（EMA））对药物中GTI的控制提出了严格要求^［9］^，其中ICH M7指导原则明确了此类杂质的鉴定、评估和限量标准，通常要求其含量控制在毒理学关注阈值（TTC）以下。WHT1408-Q2H是由偶氮二羧酸二异丙酯通过光延反应过程生成的，是克唑替尼原料药合成工艺的一个副反应产物。这个杂质具有肼的警示结构（见图1），在体内可能主要通过代谢活化生成碳正离子和碳氧中心自由基等活性较强的中间体，再与DNA碱基发生烷基化反应，是一种潜在的GTI^［10］^。然而，目前尚未有针对WHT1408-Q2H杂质检测的方法，因此，开发一种快速、简便的方法对其进行高效检测至关重要。

**图1:**
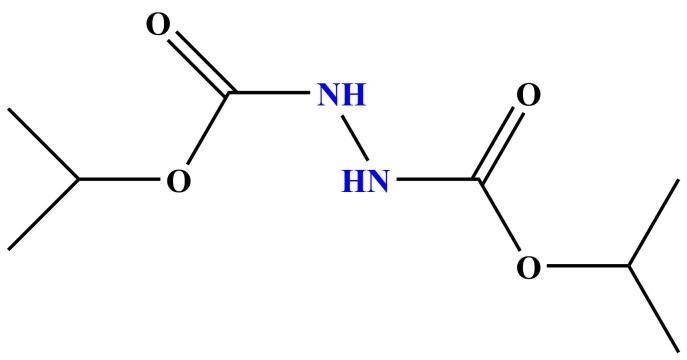
基因毒性杂质WHT1408-Q2H的警示结构

本文建立了一种高效液相色谱-电喷雾电离串联质谱（HPLC-ESI-MS/MS）方法，用于检测克唑替尼原料药中潜在的WHT1408-Q2H，并进行了系统的方法学验证。由于缺乏相关安全性研究数据，因此根据ICH M7以及《中国药典》2020年版四部通则9306遗传毒性杂质控制指导原则^［11］^，采用TTC可接受摄入值计算限度：本品最大日剂量为0.5 g，其治疗期为1~10年，则该杂质允许最大日摄入量10 µg/d，其限度为20 ng/mg。

## 1 实验部分

### 1.1 仪器、试剂与材料

Agilent 6470 高效液相色谱-三重四极杆质谱联用系统（Agilent 公司，美国）；XS205十万分之一天平（Mettler Toledo 公司，瑞士）。甲酸、乙腈（HPLC级，上海麦克林生化科技股份有限公司）；克唑替尼原料药（批号：WHT1408-Y-190804、WHT1408-Y-221019、WHT1408-Y-221022、WHT1408-Y-221028，江苏万邦生化医药集团有限责任公司）；WHT1408-Q2H（批号：H1821918，含量：97%，上海阿拉丁生化科技股份有限公司）。

### 1.2 实验条件

#### 1.2.1 对照品溶液的制备

精密称取WHT1408-Q2H对照品20.00 mg，以水-乙腈（30∶70，v/v）为稀释剂，制成质量浓度为1 µg/mL的对照品储备液。精密量取1 mL对照品储备液置于量瓶中，用稀释剂稀释至刻度，摇匀，得到质量浓度为20 ng/mL的对照品溶液。

#### 1.2.2 样品前处理

取克唑替尼原料药（批号：WHT1408-Y-190804）约50 mg，精密称定，置于50 mL量瓶中，加入水-乙腈（30∶70，v/v）溶解并稀释至刻度，摇匀即得，作为供试品溶液。

#### 1.2.3 色谱条件

色谱柱为Agilent Eclipse XDB C8色谱柱（150 mm×4.6 mm，3.5 µm），柱温为40 ℃，流动相为0.1%甲酸水溶液（A）-0.1%甲酸乙腈溶液（B），梯度洗脱程序如下：0~10.0 min，10%B~95%B；10.0~10.1 min，95%B～10%B；10.1~15.0 min，10%B。流速为0.4 mL/min，进样量为5 μL。

六通阀切换设置：时间为5.0~8.0 min时，流动相进入废液，其余时间流动相进入质谱检测。

#### 1.2.4 质谱条件

电喷雾正离子化（ESI^+^），多反应检测（MRM）模式，鞘气温度为250 ℃，鞘气流速为11 L/min，喷嘴电压为500 V，雾化器压力为241 325 Pa，毛细管电压为4 000 V，干燥气流速为5 L/min，干燥气温度为300 ℃；WHT1408-Q2H的检测离子对为*m*/*z* 205.3（母离子）、*m*/*z* 121.0（子离子）， 裂解电压为65 V，碰撞能量为5 eV。

## 2 结果与讨论

### 2.1 实验条件考察

#### 2.1.1 色谱条件的优化

流速的考察：本研究对流动相流速进行了考察，分别评估了0.8 mL/min与0.4 mL/min两种流速条件下WHT1408-Q2H的色谱响应特性。结果显示，流速为0.8 mL/min时，没有出现目标物峰（图2a）；当流速调整为0.4 mL/min时，目标杂质的色谱响应强度明显增强（图2b），且呈现多峰状态。基于此优化结果，为确保分析方法具有最佳的检测灵敏度，最终确定采用0.4 mL/min作为方法开发的优选流速条件。

**图2:**
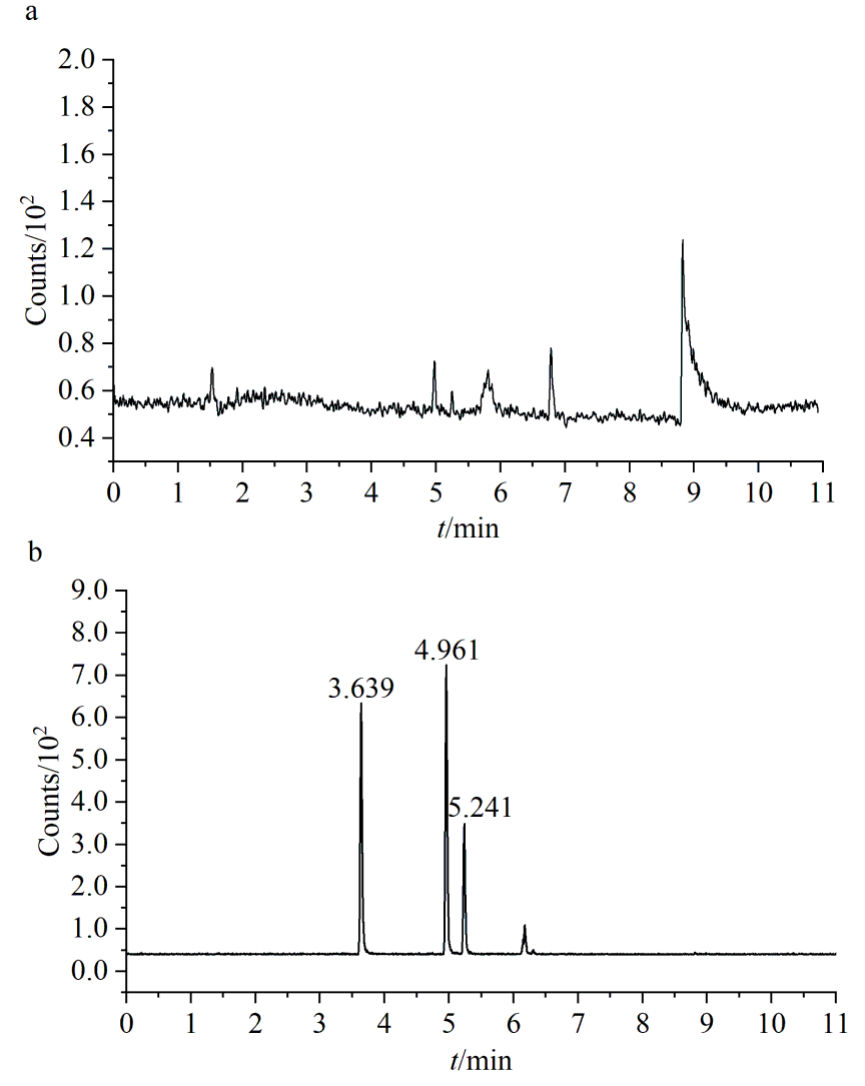
以（a）0.8 mL/min与（b）0.4 mL/min作为流速时对照品溶液的TIC色谱图

洗脱程序的考察：在流速为0.4 mL/min条件下，杂质WHT1408-Q2H呈现多响应质谱峰，经结构解析确认系其烯醇-酮式互变异构现象所致^［12］^。在WHT1408-Q2H结构中，由于羰基的作用，*α*-H变得非常活泼，呈现出酸性特性，因此会以烯醇式和酮式之间的平衡状态存在，并不断发生互变。相较于酮式，烯醇式的最大吸收波长向长波长方向移动，同时吸光度也有所增加。在极性方面，酮式状态缺乏亲水基团，极性非常小；而烯醇式状态引入了羟基，极性显著增大。因此在定量分析中，烯醇式极性增加，在反相色谱上的保留能力较弱，导致出峰时间较早。同时，其光谱图和响应值也可能与酮式有所不同。因此，可通过改变流动相中有机相的比例改变流动相极性、pH值来规避这种互变异构现象^［13］^。根据文献报道，降低初始有机相比例（即增大极性）可促进酮式构型稳定化。经实验验证，发现降低初始有机相比例后，杂质WHT1408-Q2H只有一个峰，保留时间为7.0 min，但此条件下供试品的主要物质的保留时间为5.5 min，且在6.3 min时完全出峰，与杂质WHT1408-Q2H分离效果差。

因此，为实现供试品与杂质峰的有效分离，本研究对梯度洗脱程序进行了进一步的系统性优化。通过调节有机相比例，最终确定的洗脱条件如1.2.3节所示。优化后的对照品溶液色谱图如图3所示，WHT1408-Q2H的保留时间为9.1 min。优化后的供试品溶液色谱图如图4所示，保留时间为6.5 min，在约7.2 min完全出峰，说明WHT1408-Q2H与供试品分离度改善。

**图3:**
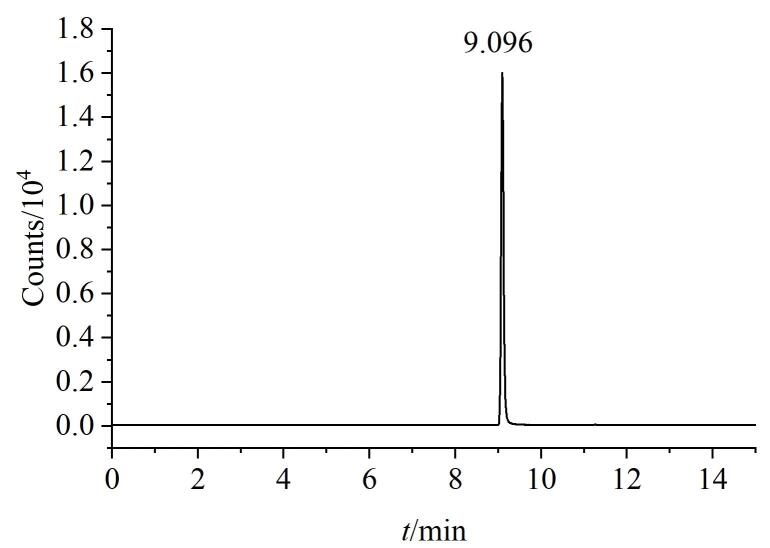
优化条件下WHT1408-Q2H对照品溶液的TIC色谱图

**图4:**
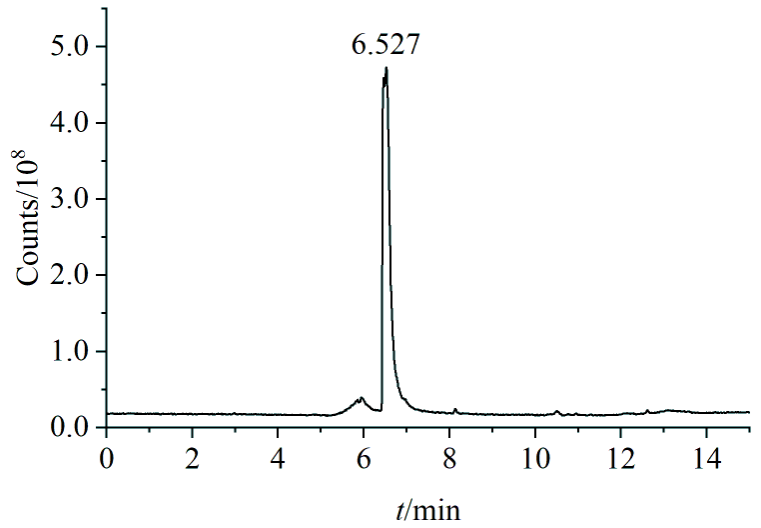
优化条件下供试品溶液的TIC色谱图

样品盘温度的考察：样品盘控温过程中可能产生冷凝水，若冷凝水积聚未能及时排出，仪器检测系统会触发报错机制导致分析序列中断，从而增加实验异常风险。为此，本研究对样品在室温条件下的稳定性（分别放置0、2、4、7、9 h）进行了系统考察。结果表明，对照品及供试品溶液的色谱峰面积均未呈现显著性变化（对照品峰面积的RSD为9.7%，供试品溶液的RSD为7.0%，均<10%），证明杂质WHT1408-Q2H对照品溶液与供试品溶液在室温条件下具有良好的化学稳定性。基于此实验结果，建议取消样品盘控温设置，且该操作不会对分析结果的准确性产生显著影响

#### 2.1.2 质谱条件的优化

采用电喷雾电离源（ESI），分别在正、负离子模式下对目标化合物WHT1408-Q2H（相对分子质量204.23）进行母离子扫描。该化合物在正离子模式下易形成*m*/*z* 205.3的准分子离子峰［M+H］^+^，故选择正离子模式进行后续质谱参数优化。

首先在选择离子监测（SIM）模式下对裂解电压进行系统优化。随后采用MRM模式，通过梯度测试（50~90 V，步长5 V）考察裂解电压对离子传输效率的影响。基于信号响应强度，最终确定65 V为最优裂解电压。

在产物离子扫描模式下，对特征碎片离子进行筛选及碰撞能量（CE）优化。选择*m*/*z* 205.3/121.0作为定量离子对，通过对比不同碰撞能量（3、5、7、10 eV）下的离子丰度，确定5 eV为最佳碰撞能量。最佳质谱条件如1.2.4节所示，WHT1408-Q2H杂质的二级质谱图如图5所示。

**图5:**
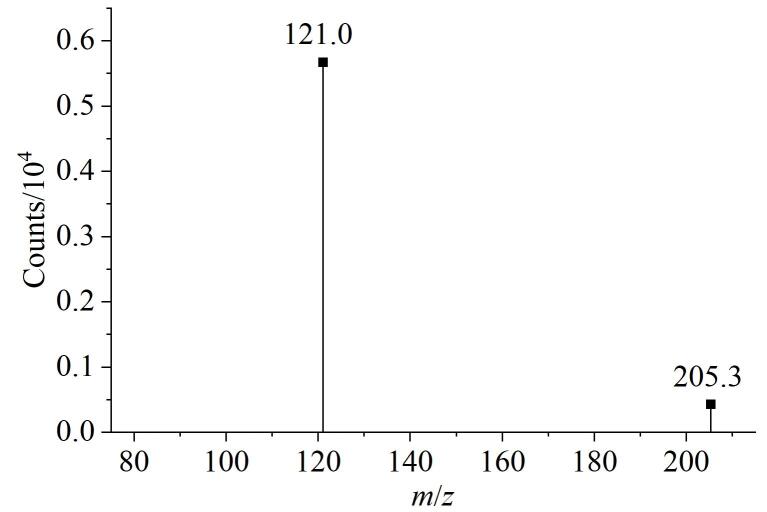
WHT1408-Q2H的二级质谱图

### 2.2 方法学验证

#### 2.2.1 专属性

精密进样空白溶剂、对照品溶液及供试品溶液各5 µL。通过对照品溶液确证杂质WHT1408-Q2H的保留时间为9.151 min。空白溶剂及供试品溶液色谱图中，目标杂质峰保留时间处未见显著干扰峰（*S*/*N<*3），表明该方法对目标杂质具有良好的分析专属性，符合方法学验证要求^［10］^。相关色谱图见图6。

**图6:**
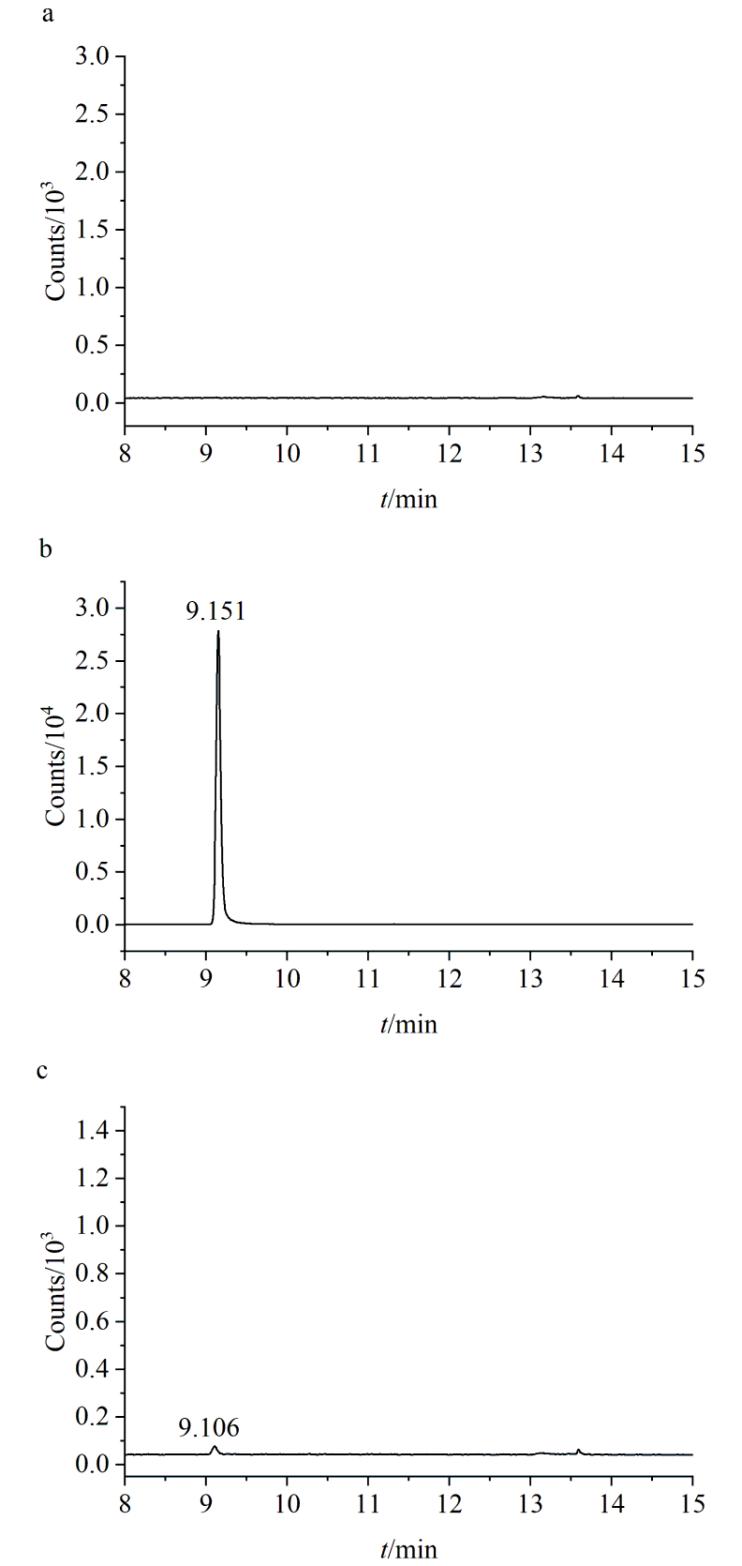
（a）空白溶剂、（b）对照品溶液和（c）供试品溶液的TIC图

#### 2.2.2 线性关系

精密量取WHT1408-Q2H对照品储备液适量，稀释成2、6、10、20、30、40 ng/mL系列标准溶液，进样分析。以峰面积为*y*，质量浓度（ng/mL）为*x*，不加权重拟合线性校正曲线，建立线性回归方程：*y*=6.320×10^3^ *x-*4.572×10^3^，*r*=0.999 9。结果表明WHT1408-Q2H的峰面积与其质量浓度在2~40 ng/mL范围内呈现良好的线性关系。

#### 2.2.3 检出限和定量限

精密量取1.2.1节的对照品储备溶液，用稀释剂逐级稀释，按照*S*/*N*≥3和≥10分别计算检出限和定量限。结果表明，杂质WHT1408-Q2H的检出限为0.396 9 ng/mL（即0.396 9 ng/mg），相当于限度浓度^［10］^（20 ng/mg）的2%；定量限为1.984 6 ng/mL（即1.984 6 ng/mg），相当于限度浓度的10%。6次定量限平行试验中，峰面积的RSD（*n*=6）为0.73%，不大于15%，重复性良好，且*S*/*N*均大于10，说明本方法的最低检测能力满足使用要求。

#### 2.2.4 精密度

取WHT1408-Q2H对照品溶液，连续进样6次，WHT1408-Q2H峰面积的RSD（*n*=6）为0.49%，表明本方法的仪器精密度良好。

#### 2.2.5 稳定性

取对照品溶液于进样瓶中，按照实验条件分别放置0、2、5、8、11、14、16、19 h后进样测定。结果显示WHT1408-Q2H峰面积的RSD（*n*=8）为1.0%，说明该条件下WHT1408-Q2H在19 h内稳定性良好。

#### 2.2.6 回收率

取克唑替尼原料药约50 mg，精密称取9份（低、中、高水平各3份），分别精密加入对照品储备液各0.5、1、1.5 mL，用稀释剂溶解并稀释至刻度，得到加标回收试验用供试品溶液，分别进样分析。结果如表1显示，克唑替尼原料药的加标回收率为95.6%~102.7%，RSD（*n=*3）为0.4%~0.7%，不大于15%，符合药典规定，说明本方法的准确度良好。

**表1:** 克唑替尼中杂质WHT1408-Q2H的加标回收率（*n*=3）

Added/（ng/mL）	Recovery/%	RSD/%
9.9231	95.6	0.7
19.8462	100.2	0.4
29.7693	102.7	0.4

#### 2.2.7 实际样品检测

取3批克唑替尼原料药（批号：WHT1408-Y-221019、WHT1408-Y-221022、WHT1408-Y-221028），按1.2.2节方法制备供试品溶液，按已验证的方法进样测定。以外标法计算WHT1408-Q2H的含量，3批克唑替尼原料药样品中均未检出杂质WHT1408-Q2H，表明克唑替尼原料药生产企业在生产工艺中对基因毒性杂质控制良好。

## 3 结论

本文建立了HPLC-ESI-MS/MS测定克唑替尼原料药中基因毒性杂质WHT1408-Q2H的方法，该方法展现出高灵敏度、准确度以及良好的重现性，符合ICH M7指导原则的要求。此外，该方法的建立不仅为克唑替尼中基因毒性杂质的检测提供了有力的工具，也为其他药物中类似杂质的检测提供了参考和借鉴。随着药物分析技术的不断发展，该方法有望在未来得到更广泛的应用和推广，为药物质量控制和安全性评价提供更有力的支持。
